# Changes in self-reported and accelerometer-measured physical activity among pregnant TRICARE Beneficiaries

**DOI:** 10.1186/s12889-022-14457-2

**Published:** 2022-11-07

**Authors:** Kinsey Pebley, Gregory Farage, Marion E. Hare, Zoran Bursac, Aline Andres, Sultana Mubarika Rahman Chowdhury, G. Wayne Talcott, Rebecca A. Krukowski

**Affiliations:** 1 Department of Psychology, University of Memphis, Memphis, Tennessee USA; 2 Department of Preventive Medicine, University of Tennessee Health Science Center, Memphis, Tennessee USA; 3 Department of Biostatistics, Florida International University, Miami, Florida USA; 4 University of Arkansas for Medical Sciences and Arkansas Children’s Nutrition Center, Little Rock, Arkansas USA; 5 Wilford Hall Ambulatory Surgical Center, San Antonio, Texas USA; 6 Department of Public Health Sciences, University of Virginia, University of Virginia Cancer Center, PO Box 800765, Charlottesville, Virginia 22903 USA

**Keywords:** Physical activity, Pregnancy, Military, Randomized controlled trial, Accelerometer

## Abstract

**Background:**

Physical activity is recommended for all pregnant individuals and can prevent excessive gestational weight gain. However, physical activity has not been assessed among military personnel and other TRICARE beneficiaries, who experience unique military lifestyles. The current study assessed physical activity among pregnant TRICARE beneficiaries, both active duty and non-active duty, as measured by accelerometry and self-report data to examine potential predictors of physical activity engagement in the third trimester, and if self-report data was consistent with accelerometry data. We expected having a lower BMI, being active-duty, and having higher baseline physical activity engagement to be associated with higher physical activity at 32-weeks. We also hypothesized that accelerometry data would show lower physical activity levels than the self-reported measure.

**Methods:**

Participants were 430 TRICARE adult beneficiaries (204 Active Duty; 226 non-Active Duty) in San Antonio, TX who were part of a randomized controlled parent study that implemented a stepped-care behavioral intervention. Participants were recruited if they were less than 12-weeks gestation and did not have health conditions precluding dietary or physical activity changes (e.g., uncontrolled cardiovascular conditions) or would contribute to weight changes. Participants completed self-report measures and wore an Actical Activity Monitor accelerometer on their wrist to collect physical activity data at baseline and 32-weeks gestation.

**Results:**

Based on the accelerometer data, 99% of participants were meeting moderate physical activity guidelines recommending 150 min of moderate activity per week at baseline, and 96% were meeting this recommendation at 32-weeks. Based on self-report data, 88% of participants at baseline and 92% at 32-weeks met moderate physical activity recommendations. Linear regression and zero-inflated negative binomial models indicated that baseline physical activity engagement predicted moderate physical activity later in pregnancy above and beyond BMI and military status. Surprisingly, self-reported data, but not accelerometer data, showed that higher baseline activity was associated with decreased vigorous activity at 32-weeks gestation. Additionally, self-report and accelerometry data had small correlations at baseline, but not at 32-weeks.

**Conclusions:**

Future intervention efforts may benefit from intervening with individuals with lower pre-pregnancy activity levels, as those who are active seem to continue this habit.

**Trial Registration:**

The trial is registered on clinicaltrials.gov (NCT 03057808).

## Background

Physical activity is recommended for all those who are pregnant [1] and can prevent excessive gestational weight gain (GWG) [2], as well as promote other health benefits [2, 3]. However, physical activity may be particularly important among pregnant active-duty United States (U.S.) military personnel since they are required to pass physical fitness tests at 12 months postpartum. Failure to successfully meet physical fitness guidelines may result in discharge from military service [4]. Guidelines are provided about personnel needing to maintain “physical readiness,” or the ability to meet physical demands of duty or combat [5] and how much weight pregnant personnel should gain over the course of their pregnancy [6], but there is limited information regarding how pregnant individuals covered under TRICARE meet these guidelines and maintain physical activity in order to maintain “physical readiness” during pregnancy.

Research among civilians has shown that levels of physical activity are lower during pregnancy compared to reported pre-pregnancy activity [7], and most pregnant individuals do not receive information about exercising during their prenatal care [8]. However, many studies of physical activity in pregnant individuals rely on self-report, such as the International Physical Activity Questionnaire (IPAQ) [9–11]. Pregnancy-specific self-report measures of physical activity have been created (e.g., Pregnancy Physical Activity Questionnaire [PPAQ]), but have still been found to overestimate the amount of physical activity [12] when compared to accelerometry data, which can provide real-time and more objective data. In addition, there are few studies that examine changes in physical activity over the course of pregnancy and none, of which we are aware, that have assessed physical activity changes over the course of pregnancy by accelerometer among active-duty personnel. Active-duty personnel may be more likely to be physically active during pregnancy compared to civilians given their job requirements.

The current study aimed to assess physical activity levels among pregnant TRICARE beneficiaries, both active-duty and non-active-duty (i.e., partners or dependents of active-duty personnel), as measured by accelerometry and self-report data to examine potential predictors of physical activity engagement in the third trimester, when activity is expected to be lower [7]. We also aimed to determine if self-report data provided by participants was consistent with their accelerometry data. We expected having a lower BMI, being active-duty, and having higher baseline physical activity engagement to be associated with higher physical activity at 32-weeks. We also hypothesized that accelerometry data would show lower physical activity levels than the self-reported measure.

## Methods

### Participants and procedures

Participants were 430 TRICARE beneficiaries ages 18 years or older living in San Antonio, TX, USA who were part of a randomized controlled trial parent study that implemented a stepped-care behavioral intervention program based on the Look AHEAD (Action for Health in Diabetes) intensive lifestyle intervention [13–15]. Rationale and methodology plans are described elsewhere [16]. Briefly, the study provided interventions for physical activity and diet modifications to prevent excessive GWG and/or postpartum weight loss while also managing the challenges that accompany military lifestyles (e.g., deployment, base environment). Participants were recruited if they were less than 12-weeks gestation and were excluded from the study if they had health conditions that would preclude dietary or physical activity changes (e.g., uncontrolled cardiovascular conditions) or would contribute to weight changes (e.g., uncontrolled thyroid conditions). Additionally, potential participants taking medications that may impact weight, recent bariatric surgery or history of significant weight loss (more than 4.5 kg in the past 3 months), unmanaged psychiatric conditions, and smoking were exclusion criteria. Participants were recruited from medical clinics on military bases in the U.S. and completed a screening visit and received medical clearance before being randomized to a study condition.

Participants could be randomized into one of three conditions: a GWG intervention (occurring during pregnancy to address management of weight gain from enrollment in the first trimester to delivery), a post-partum weight loss (PPWL) intervention (occurring from 6 weeks post-partum to 12 months post-partum to address weight loss in the post-partum period), or a GWG + PPWL intervention, and the outcomes of this trial are described elsewhere [17]. The interventions were delivered via telephone and electronic scales to reduce participant burden given the challenges of accessing face-to-face support within military families, similar to the delivery of the Look AHEAD intensive lifestyle intervention among military personnel in the Fit Blue study [18, 19]. Informed consent was obtained from all participants. All study procedures were approved by the 59^th^ Medical Wing Institutional Review Boards. All methods were carried out in accordance with Declaration of Helsinki. Study recruitment occurred from February 2017 to October 2020. Physical activity data were collected from February 2017 to March 2021, and follow-up data was collected by March 2021. The trial ended after recruitment and data collection were completed.

### Measures

#### Demographics and physical measurements

At their screening visit, participants self-reported demographics, including age, race, ethnicity, and previous live births. Height was measured at the screening visit or self-reported during the COVID-19 pandemic. Weight was measured at each visit using a calibrated digital scale while the participant was wearing light clothing and no shoes or cellularly transmitted to the study team using a BodyTrace smart scale during the COVID-19 pandemic. The screening visit weight was used to calculate pre-pregnancy body mass index (BMI), given past literature showing that early first trimester BMI is a good proxy for pre-pregnancy BMI [20].

#### Physical activity

Participants also completed a self-report measure of physical activity: the International Physical Activity Questionnaire (IPAQ) at baseline and 32-weeks gestation. This measure has previously been used in studies related to pregnant individuals [9, 11]. Moderate and vigorous activity minutes were added together to create scores for physical activity engagement.

Participants also wore an Actical Activity Monitor accelerometer on their wrist to provide objective physical activity data. These measurements were obtained at baseline and 32-weeks gestation. It is important to note that, due to the COVID-19 pandemic, accelerometer data was only captured at baseline and 32 weeks up until March 2020, as it was unclear if providing accelerometers (and having staff members and participants touch objects handled by others) was safe once COVID-19 was initially identified. Thus, there is greater missing data for the accelerometry. Specifically, 38% of participants had missing accelerometer data at baseline and 57% at 32-weeks gestation. For self-reported physical activity, 1% were missing these data at baseline and 13% at 32-weeks follow-up.

The activity data were retrieved from the Actical activity monitoring devices (Philips Respironics Co. Inc., Bend, OR) using the Actical Software. Actical accelerometers were set up to record accumulated activity counts every minute, collecting 1440 samples daily. Physical activity was assessed on a minimum of three days and a maximum of 5 days, including at least 1 weekend day and 2 weekdays, consistent with previous research [21, 22]. Days with less than 24 h of recorded activity were excluded [21, 22]. The information extracted from the Actical accelerometer for each epoch included the time and date, activity count, number of steps, activity energy expenditure (kcals/min/kg), and the level of physical activity. Total activity counts per day were calculated, and the days where the total activity count was less than 250 counts were also excluded. Finally, the time spent in each of the four levels of physical activity (i.e., sedentary, light, moderate and vigorous) was reported according to the following cutoff points [23]. The level of activity was tagged as sedentary when the average activity count for 3 successive minutes was less than 50 counts/min in each epoch. All epochs lower than 600 counts/min but not labeled as sedentary were marked as light. However, if the epoch activity count was higher than 600 counts/min, the activity energy expenditure was compared with another set of kcals/min/kg cutoff points [23]. In this case, if the activity energy expenditure was less than 0.031 kcal/min/kg, the epoch was labeled as light. If the activity energy expenditure was more than 0.031 kcal/min/kg and less than 0.083 kcal/min/kg, the epoch was marked as moderate; otherwise, the epoch was tagged as vigorous, with an activity energy expenditure greater than 0.083 kcal/min/kg.

### Data analysis

All data analyses were carried out with SAS/STATv15.2. Outcome measures of the moderate and vigorous physical activity minutes were examined for distributional normality using Kolmogorov–Smirnov test, summary statistics and distributional visualizations, and the average number of daily minutes for each intensity level was used in the final models. A Bland–Altman difference plot was used to assess the agreement between accelerometry and self-reports for moderate and vigorous activity at baseline and 32-weeks [24, 25]. Descriptive statistics included frequencies and proportions, as well as means and standard deviations for discrete and continuous variables, respectively. They were generated for the overall sample and by military status. Comparisons by military status were performed with two-sample t-test or chi-square test, respectively. Based on observed outcome distributions, both self-reported and accelerometry moderate physical activity were modeled with a multivariable linear regression model. In contrast, we applied zero-inflated negative binomial model for self-reported and accelerometry vigorous physical activity due to data dispersion and excess zero mass. Models controlled for baseline activity, intervention group, previous live birth, and BMI category. These variables were included in the model given their potential influence on activity levels during pregnancy. For example, previous live births may indicate someone who may have more knowledge about pregnancy and thus what to expect physically.

## Results

### Participant characteristics

Participant characteristics are displayed in Table 1. About 68% identified as White, 15% identified as Black or African American, and 17.2% identified with other racial identities. At baseline, 32.8% were of healthy BMI, 40.0% had overweight, and 27.2% had obesity. Two-hundred and four participants were active-duty and 226 were other TRICARE beneficiaries. Active-duty and other TRICARE beneficiaries were significantly different in racial identity, BMI, and number of previous live births, but not age or intervention assignment.

**Table 1:** Participant characteristics

	**Active-Duty (*n* = 204)**	**Other TRICARE Beneficiary (*n* = 226)**		**Overall (*N* = 430)**
	***N* (%)**	***N* (%)**	***p***	***N* (%)**
**Race**			0.001	
Black or African American	43 (21.1%)	21 (9.3%)		64 (14.9%)
White	134 (65.7%)	158 (69.9%)		292 (67.9%)
Other racial groups	27 (13.2%)	47 (20.8%)		74 (17.2%)
**Body Mass Index (BMI)**			0.001	
Healthy	65 (31.9%)	76 (33.6%)		141 (32.8%)
Overweight	98 (48.0%)	74 (32.7%)		172 (40.0%)
Obese	41 (20.1%)	76 (33.6%)		117 (27.2%)
**Previous Live Birth**			0.03	
Yes	102 (50.0%)	137 (60.6%)		239 (55.6%)
No	102 (50.0%)	89 (39.4%)		191 (44.4%)
**Intervention Assignment**			0.49	
GWG-only/GWG + PPWL	140 (68.6%)	148 (65.5%)		288 (67%)
PPWL only	64 (31.4%)	78 (34.5%)		142 (33%)
**Baseline Physical Activity**				
150 + min moderate activity – Self- Report	172 (85.2%)	203 (90.2%)	0.11	375 (87.8%)
150 + min moderate activity – Accelerometer	131 (99.2%)	130 (96.3%)	0.21	261 (97.8%)
**32 Weeks Physical Activity**				
150 + min moderate activity – Self- Report	163 (91.6%)	182 (92.4%)	0.77	345 (92%)
150 + min moderate activity – Accelerometer	89 (98.9%)	90 (94.7%)	0.21	179 (96.8%)
**Age** (*M* (*SD*))	30.6 (5.2)	30.7 (4.5)	0.89	30.6 (4.9)

*M* Mean, *SD* Standard deviation; *GWG* Gestational weight gain, *PPWL* Post-partum weight loss. a = Denominators do not reflect the total Ns listed, but rather the number of participants with complete data

The average daily minutes engaging in moderate and vigorous physical activities at both time points for both self-report and accelerometry data are displayed in Table 2. At baseline, participants, overall, were averaging 111.0 (*SD* = 63.9) minutes of moderate physical activity each day based on accelerometry data and 133.8 (*SD* = 130.7) minutes of moderate physical activity each day based on self-reported data. This level of physical activity was maintained at 32-weeks gestation.

**Table 2:** Average engagement in moderate and vigorous physical activity at each time period

	**Active Duty**		**Other TRICARE Beneficiary**		**Overall**	
Average Daily Physical Activity (in minutes)	**Baseline *M* (*SD*)**	**32 Weeks *M* (*SD*)**	**Baseline *M* (*SD*)**	**32 Weeks *M* (*SD*)**	**Baseline *M* (*SD*)**	**32 Weeks *M* (*SD*)**
Accelerometer	*N* = 132	*N* = 90	*N* = 135	*N* = 95	*N* = 267	*N* = 185
Moderate	109.1 (54.6)	102.2 (61.1)	112.9 (72)	117.8 (69.8)	111.0 (63.9)	110.2 (66.0)
Vigorous	0.7 (1.76)	0.18 (0.7)	0.45 (1.65)	0.36 (1.54)	0.57 (1.7)	0.27 (1.21)
Self-report	*n* = 202	*n* = 178	*n* = 225	*n* = 197	*n* = 427	*n* = 375
Moderate	130.4 (126.5)	147.6 (142.1)	136.8 (134.6)	168.9 (151.9)	133.8 (130.7)	158.9 (147.5)
Vigorous	41.7 (75)	29.9 (68.8)	20 (41.6)	47.9 (91.7)	30.2 (60.7)	39.3 (82)

*M* Mean, *SD* Standard deviation

Based on the accelerometer data, 99% of participants were meeting moderate physical activity guidelines recommending 150 min of moderate activity per week [26] at baseline, and 96% were meeting this recommendation at 32-weeks. Based on self-report data, about 88% of participants at baseline and 92% at 32-weeks met moderate physical activity recommendations. It is also important to note that there were no significant differences in physical activity at baseline or follow-up between intervention groups for self-report and accelerometer data.

### Self-report and accelerometer agreement

**Fig. 1:**
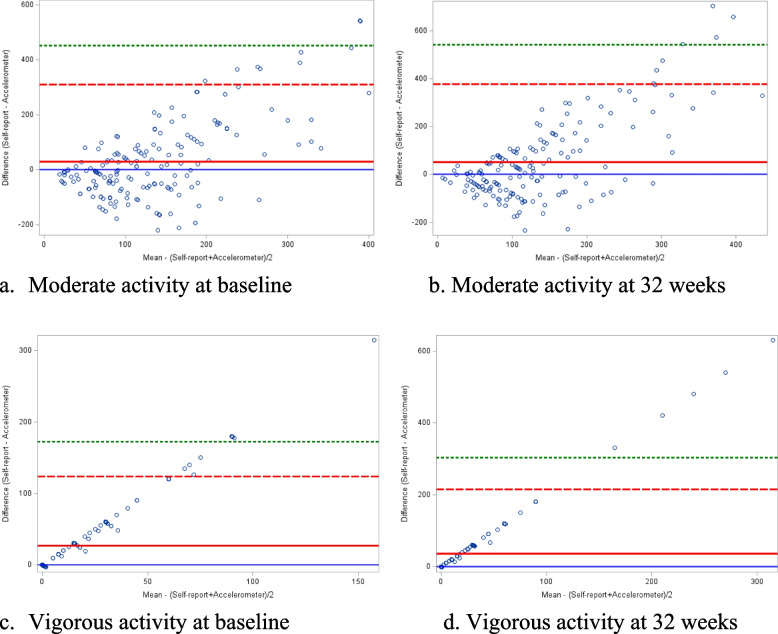
Agreement between self-report and accelerometer data **a** Moderate activity at baseline **b**. Moderate activity at 32 weeks. **c** Vigorous activity at baseline. **d**. Vigorous activity at 32 weeks

Figure 1 depicts the agreement between self-reported and accelerometer data. Self-reported and accelerometer-measured baseline moderate (*r* = 0.20, *p* = 0.01) and vigorous activity (*r* = 0.16, *p* = 0.04) had small statistically significant correlations. At 32-weeks gestation, self-reported and accelerometer-measured moderate (*r* = 0.05, *p* = 0.48) and vigorous activity (*r* = 0.05, *p* = 0.52) were not significantly correlated.

### Predicting physical activity at 32-Weeks gestation

Table 2 displays the average amount of daily moderate and vigorous activity across groups from both self-report and accelerometry data, while Table 3 displays results from final adjusted models for both self-report and accelerometry data. In models using self-report data, baseline physical activity was positively associated with moderate activity (*B* = 0.58, *SE* = 0.07, *p* < 0.0001) and negatively associated with vigorous physical activity at 32-weeks (*B* = -0.02, *SE* = 0.01, *p* = 0.0002). Higher baseline activity was associated with increased odds of zero reported vigorous activity (*B* = 0.01, *SE* = 0.002, *p* = 0.02). BMI category and military status were not significantly associated with 32-week activity (*p* > 0.05).

**Table 3:** Regression results predicting moderate activity at 32 weeks

	**Predicting Self-Reported 32-Week Moderate Physical Activity**	**Predicting Self-Reported 32-Week Vigorous Physical Activity**				
		**Logit**			**NB**	
	***B(SE)***	***p***	***B(SE)***	***p***	***B***	***p***
**Military Status**						
Active Duty	17.2 (20.5)	0.40	-0.27 (0.26)	0.30	0.63(0.37)	0.09
Other TRICARE beneficiary	Reference					
**Body Mass Index**						
Normal Weight	Reference					
Overweight	16.1 (27.1)	0.55	0.19 (0.31)	0.52	0.13 (0.4)	0.76
Obese	-2.7 (23.4)	0.91	0.47 (0.34)	0.17	-0.02 (0.46)	0.97
**Baseline activity**	0.58 (0.07)	< 0.0001	0.01 (0.002)	0.02	-0.02 (0.01)	0.0002
	**Predicting Accelerometer-Measured 32-Week Moderate Physical Activity**	**Predicting Accelerometer-Measured 32-Week Vigorous Physical Activity**				
		**Logit**			**NB**	
	***B(SE)***	***p***	***B(SE)***	***p***	***B***	***p***
**Military Status**						
Active Duty	-15.4 (8.8)	0.08	-2.7(0.81)	0.0008	-25.1(121,081)	1.00
Other TRICARE beneficiary	Reference					
**Body Mass Index**						
Normal Weight	Reference					
Overweight	6.8 (10)	0.4959	2.06 (0.83)	0.01	1.7 (2.1)	0.41
Obese	-1.8 (11.6)	0.8774	-0.5 (0.95)	0.59	-22.6 (118,166)	1.00
**Baseline activity**	0.47 (0.07)	< 0.0001	0.62 (0.22)	0.006	0.15 (0.28)	0.60

Models controlled for baseline activity, age, intervention group, and previous live birth. *SE* Standard error, *NB* Negative binomial. Zero-inflated negative binomial model consists of logistic model and negative binomial model

In models using accelerometry data, baseline activity also predicted engagement in moderate activity at 32-weeks (*B* = 0.47, *SE* = 0.07, *p* < 0.0001), but not vigorous activity at 32-weeks (*p* > 0.05). BMI category and military status were not significantly associated with 32-week activity (*p* > 0.05).

## Discussion

The current study found that baseline physical activity engagement predicts engagement in moderate physical activity later in pregnancy above and beyond BMI category or military duty status. In other words, past behavior predicts future behavior when it comes to moderate physical activity engagement during pregnancy. This finding is consistent with past research demonstrating that women with higher activity levels prior to pregnancy are more likely to engage in higher levels of physical activity during pregnancy (see review by Gaston & Cramp) [7]. Additionally, self-reported data showed that higher baseline activity was associated with decreased vigorous activity at 32-weeks gestation, although this was not corroborated using accelerometer data. This finding may indicate that individuals who are very physically active or engaging in higher intensity physical activities early in pregnancy may perceive a shift later in pregnancy to less intense physical activity.

The current study also found that majority of participants (88% +) in the sample were meeting or exceeding the physical activity guidelines of 150 or more minutes of moderate activity per week. Further, participants were approaching the 150-min recommendation in a given day. Another study of pregnant individuals assessing self-reported physical activity rates over the course of pregnancy found that only about 23.4% of participants achieved 150 min of physical activity during the week [27]. Our results are surprising given that so many participants were exceeding physical activity guidelines even during their third trimester. It is also notable that both active-duty and non-active-duty beneficiaries were engaging in these high levels of physical activity. We expected active-duty personnel to be much more active given that they must meet physical fitness standards to maintain their position in the military, while other TRICARE beneficiaries are not expected to maintain specific fitness standards. It may be the case that exposure to military lifestyles impacted the physical activity of both groups. However, more research is needed to better understand why physical activity is high among these groups and how to best support its maintenance.

Additionally, self-report and accelerometry data had small correlations at baseline but were not significantly correlated at 32-weeks gestation. Consistent with past studies [12, 28], self-reported activity was typically higher than accelerometer data. One potential explanation for the discrepancy in self-report data, aside from recall bias, may be that the perceived exertion when engaging in physical activity increases as pregnancy progresses, and what is considered inactive pre-pregnancy may be considered active during pregnancy [29, 30]. It also may be due to the cutoff points for moderate and vigorous activity being based on non-pregnant samples, and different cutoffs may be needed to adequately assess activity during pregnancy given the changes they are experiencing across gestation. Differences may also be attributable, in part, to the type of accelerometer worn in this study (i.e., wrist worn). One previous study compared a waist-worn accelerometer, a thigh-worn accelerometer, and self-report measures of sedentary time across three trimesters. Results indicated that the accelerometers reported comparable sedentary time, but only had moderate agreement related to physical activity. Future research assessing the validity of different ways to measure physical activity in a way that considers the unique and changing needs of pregnant individuals are needed.

### Strengths and limitations

Despite the strengths of the study, including the range of BMI categories represented and the novelty of the research question, there are some limitations to consider. Limitations of the study include that accelerometry data collection was stopped once the COVID-19 pandemic began due to the uncertainty at the start of the pandemic as to how COVID-19 was transmitted; thus, there is more missing data with the accelerometry measurement. Additionally, the current study was conducted among TRICARE beneficiaries, and may not generalize to other pregnant individuals not covered by TRICARE. Lastly, participants who chose to participate in this study related to health and weight may have been particularly motivated to engage in positive health behaviors such as physical activity. Further research is needed to determine if active-duty and other TRICARE beneficiaries who are not part of health intervention research also engage in similar levels of physical activity.

## Conclusions

Overall, active-duty and non-active-duty TRICARE beneficiaries are largely exceeding physical activity guidelines before pregnancy and during pregnancy, even during the third trimester. Baseline activity also seems to be the best predictor of activity during pregnancy. Future intervention efforts may benefit from targeting individuals with lower pre-pregnancy activity levels regardless of BMI or military status to increase subsequent activity, as those who are active seem to continue this habit.

## Data Availability

The datasets used and/or analyzed during the current study are available from the corresponding author on reasonable request.
